# Evaluation of Bioactive Glass and Low Viscosity Resin as Orthodontic Enamel Sealer: An In Vitro Study

**DOI:** 10.3390/jfb13040191

**Published:** 2022-10-17

**Authors:** Abdullah Al Shehab, Ahmed Samir Bakry, Robert Hill, Fahad Faiz Alsulaimani, Mona Aly Abbassy

**Affiliations:** 1 Department of Orthodontics, Faculty of Dentistry, King Abdulaziz University, Jeddah 21589, Saudi Arabia; 2 Restorative Dentistry Department, Faculty of Dentistry, King Abdulaziz University, Jeddah 21589, Saudi Arabia; 3 King Fahd Medical Research Center, King Abdulaziz University, Jeddah 21589, Saudi Arabia; 4 Conservative Dentistry Department, Faculty of Dentistry, Alexandria University, Alexandria 21568, Egypt; 5 Dental Physical Sciences Unit, Institute of Dentistry, Queen Mary University of London, London E1 4NS, UK; 6 Dental Department, Alexandria University Hospital, Alexandria University, Alexandria 21532, Egypt

**Keywords:** shear bond strength, durability, resin sealer, fluoride bioactive glass

## Abstract

The study aimed to evaluate the effect of applying fluoride bioactive glass (FBAG) and Alpha-Glaze^®^ (resin sealer) on the shear bond strength of orthodontic brackets to enamel bonded by Transbond XT, brushing–abrasion durability, and their protective effect against simulated cariogenic acidic attack. Materials include 135 extracted premolars that were divided into three groups—FBAG, Alpha-Glaze, and control. The shear bond strength test was measured using an Instron Universal Testing Machine. The brushing abrasion challenge took place with a tooth-brushing simulator. Transmitted light microscopy examinations were performed after the specimens were demineralized for 4 days. The results show that the shear bond strength values of the three groups did not report any statistically significant differences: FBAG (28.1 ± 5.5 Mpa), Alpha-Glaze (32.5 ± 7.4 Mpa), and control (30.7 ± 6.5 Mpa) *p* < 0.05. The Adhesive Remenant Index (ARI) study showed chipping of enamel in 6.6% of Alpha-Glaze and control specimens and 40% of specimens had their enamel surface covered with resin. Furthermore, 30% of the FBAG and 100% of the Alpha-Glaze sealer specimens resisted the abrasion test. In conclusion, FBAG can serve as an orthodontic-sealer capable of protecting the enamel surface surrounding orthodontic brackets. However, the Alpha-Glaze sealer did not offer the capability of protecting the enamel.

## 1. Introduction

Despite the ideal outcomes acquired from orthodontic treatment, the extended treatment itself builds the risk of plaque accumulation around orthodontic fixed appliances. This increases the patient’s danger of developing caries and the likelihood of developing white spot lesions (WSLs) around fixed orthodontic brackets either buccal or lingual [1,2,3]. Throughout the orthodontic treatment, the incidences of WSLs rises 73–95% [4]. Whenever they are left untreated, white spot lesions can progress to become carious cavitations that cause esthetic problems for the orthodontic patient [5]. Therefore, it is important for the early diagnosis and prevention of these lesions [6].

Many approaches have been attempted to prevent the demineralization of enamel surrounding orthodontic brackets by various means. Methods involving the use of materials containing fluoride have been proposed to aid in the treatment of WSLs during fixed orthodontic treatment [7], but there is insufficient predictability in patient compliance [8]. Moreover, previous research shows that using fluoride-containing agents with low or high concentrations is insufficient to prevent or treat WSLs [9]. A high concentration of fluoride could significantly affect NiTi wires by affecting its mechanical properties [10,11] and may increase the risk of chronic fluoride toxicity [12]. Shielding the enamel surface with orthodontic sealants has been suggested to minimize enamel demineralization next to orthodontic brackets [4,13,14,15]. It has been reported that fluoride bioactive glass (FBAG) forms a layer containing calcium and phosphate that shows potential effects regarding remineralization and the protection of enamel next to orthodontic brackets [16,17].

Previous research shows that another bioactive glass (fluoride-free bioactive glass) withstood a brushing abrasion challenge [18] and was capable of shielding and protecting the enamel surface from erosive agents [19].

This study aimed at comparing the brushing–abrasion durability, acid resistance, and bond strength of fluoride bioactive glass (FBAG) and orthodontic Alpha-Glaze^®^ sealer applied before the bonding procedure.

The null hypotheses adopted in the current research are:

1. There will be a negative effect on shear bond strength of Transbond XT to tooth structure after the application of the fluoride bioactive glass or Alpha-Glaze sealer onto enamel surface when compared to Transbond XT.
2. There will be no differences in brushing abrasion resistance after the application of the fluoride bioactive glass when compared to the Alpha-Glaze sealer.
3. There will be no protective effect differences in protecting the teeth from demineralization after the application of the fluoride bioactive glass or Alpha-Glaze sealer when compared to the intact enamel surface.

## 2. Materials and Methods

### 2.1. Specimen Preparation

Ethical approval was obtained from the Ethical Committee at the faculty of Dentistry, King Abdul-Aziz University (Ethical #32-04-2020). For the current experiment, 135 freshly extracted teeth were utilized. All teeth were extracted for orthodontic reasons. They were sound non-carious premolars (N = 135) obtained from a patient age range of 18–25 years-old. The teeth were hand scaled of any soft tissues or calculus, then stored in freshly prepared 0.1% thymol for a maximum period of 10 days before the start of the experimental procedures. Teeth were excluded if they had cracks, demineralization, or any defects after examination by a light microscope. Teeth were divided into three equal groups: FBAG, Alpa-Glaze, and control. The crowns were separated from the roots with a low-speed diamond saw (Isomet 5000; Buehler, Lake Bluff, IL, USA). The number of samples assigned to each tested group was adopted as stated in the previous literature and according to the 80% power of test.

### 2.2. Tested Materials Application

All premolars were polished using pumice. Next, 0.10 g of bioactive glass powder was mixed on a glass slab with 2 drops of 50 wt.% phosphoric acid (prepared by diluting 85 wt.% of phosphoric acid). The resulting paste was applied to the buccal surfaces of the specimens in the FBAG group. The Alpha-Glaze group specimens had the resin sealant Alpha-Glaze (Dental Technologies Inc., Lincolnwood, IL, USA) applied on the buccal surfaces of the specimens according to the manufacturer’s instructions, while the control group did not receive any treatment at that stage. All groups were incubated for 24 h at 37 °C Table 1.

### 2.3. Transbond XT Application

The buccal surface of the three groups’ specimens were etched with 37% phosphoric acid (FineEtch, SPIDENT Co., Ltd., NamdongKongDan, Incheon, Korea) according to the recommendations of the manufacturer. Transbond XT (3M Unitek, USA) was applied according to the manufacturer’s instructions in Table 2. Metal brackets (discovery^®^ smart, by Dentaurum 0.018 slot) were bonded to the buccal surfaces.

### 2.4. Shear Bond Strength Test (SBS)

Fifteen specimens were randomly selected from each group and were subjected to a shear bond strength test using the Instron Universal Testing Machine (ElectroPlus E1000, Instron, Canton, MA, USA) with a crosshead speed of 0.5 mm/min and a 50 kg load cell [20]. The samples were situated such that the debonding force was performed parallel to the bracket base.

### 2.5. Adhesive Remnant Index (ARI)

**Figure 1:**
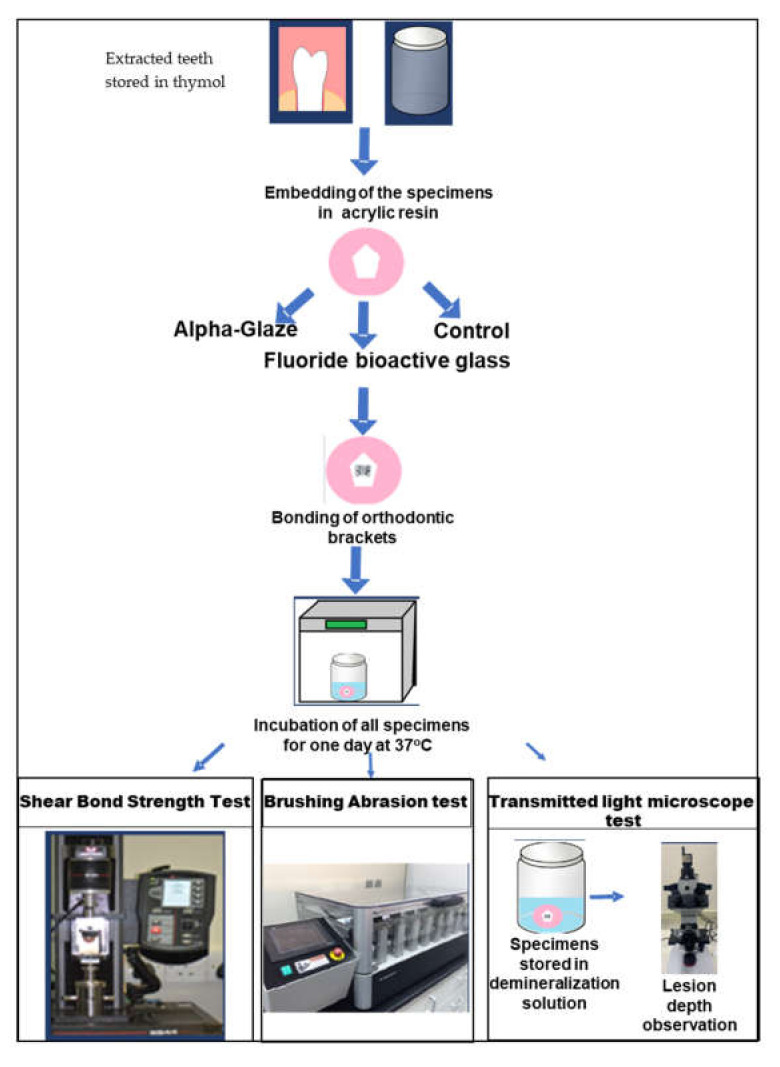
Experiments sequence.

After the shear bond strength test, the specimen’s buccal surfaces were assessed under the microscope with a magnification power of ×10. The quantity of adhesive remnant was then scored using the Adhesive Remnant Index (ARI) as shown in Table 3 and Figure 1.

### 2.6. Brushing Abrasion Challenge Test

Fifteen specimens were randomly selected from each group and were subjected to a brushing abrasion challenge test. Manual medium-bristled toothbrushes were chosen (TRISA^®^, Trisa AG, Triengen, Switzerland). Brushing experiments were performed on the buccal surfaces of the specimens with the tooth-brushing simulator. This applied 280 g to the teeth specimens with a horizontal brushing movement with a total of 7500 strokes, which corresponds to 12 months of brushing twice a day [21]. A slurry of 1:3 toothpaste (Colgate^®^; Colgate-Palmolive Co., Riyadh, Saudi Arabia) and artificial saliva was constantly cycled through the machine during the toothbrushing process Figure 1. A new brush was utilized to conduct the brushing abrasion test for each sample.

### 2.7. Transmitted Light Microscopy Examination

Fifteen specimens were randomly selected from each group. The specimens were covered completely with dark nail varnish, leaving an exposed 3 mm diameter treatment window, and then stored in demineralizing solution (2.2 mM CaCl_2_, 10 mM NaH_2_PO_4_, 50 mM acetic acid, 100 mM NaCl, 1 ppm NaF, 0.02% NaN_3_: pH 4.5) at 37 °C for 4 days. After concluding the storage period, all specimens were washed with distilled water for 1 min, embedded in low viscosity resin, and then sectioned mesiodistally to obtain 350 μm thickness slabs.

The sectioned specimens were then thinned manually with sandpapers of different grids until they reached the thickness of a maximum of 150 μm (confirmed by using a micrometer) (Figure 1). The specimens were assessed under transmitted light microscopy Figure 1.

### 2.8. Statistical Analysis

Descriptive statistics, including the mean and standard deviation (SD) for the shear bond strength and lesion depth, were subjected to a one-way ANOVA analysis followed by a Tukey post hoc test *p* < 0.05. The degree of coverage of the tested materials to the enamel surface was compared before/after the abrasion challenge with a Wilcoxon Signed-Rank test *p* < 0.05 [22]. Analysis of the results was carried out by the Statistical Package for the Social Sciences (SPSS) (SPSS 22 Inc., Chicago, IL, USA).

## 3. Results

### 3.1. Shear Bond Strength Test (SBS)

**Figure 2:**
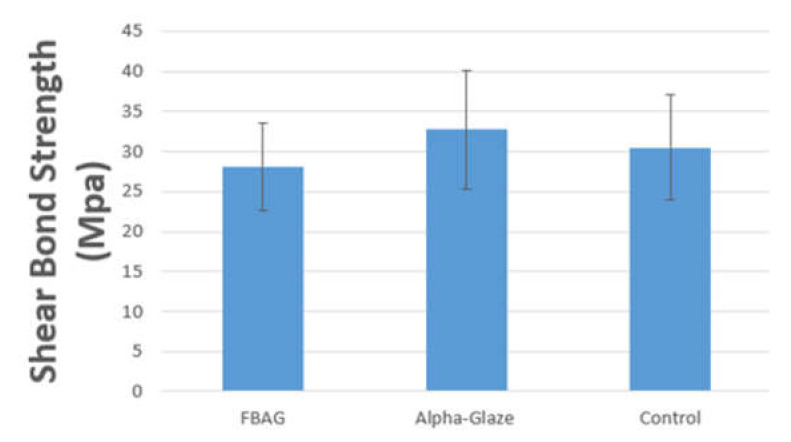
Bar chart showing the distribution of means and standard deviations of shear bond strength (SBS) in MPa.

The shear bond strength test (SBS) results are shown in Figure 2 and Table 4, the shear bond strength values of the three groups did not report any statistically significant differences *p* < 0.05.

### 3.2. Adhesive Remnant Index (ARI) Results

The ARI study showed chipping of enamel in 6.6% of the Alpha-Glaze and control groups’ specimens. Moreover, 40% of these specimens had enamel surfaces covered with resin. Specimens of the FBAG group had 40% of the surfaces not covered by any resin and 60% covered with less than 50% resin *p* < 0.05 as shown in Table 5.

### 3.3. Brushing Abrasion Challenge Test Results

As shown in Table 6, 30% of the specimens in the FBAG group and 100% of the specimens in Alpha-Glaze sealer group resisted the abrasion test.

### 3.4. Transmitted Light Microscopy Examination Results

**Figure 3:**
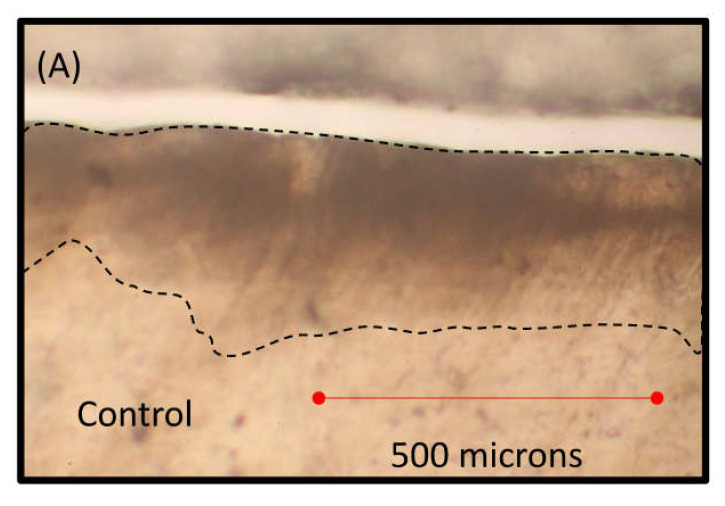

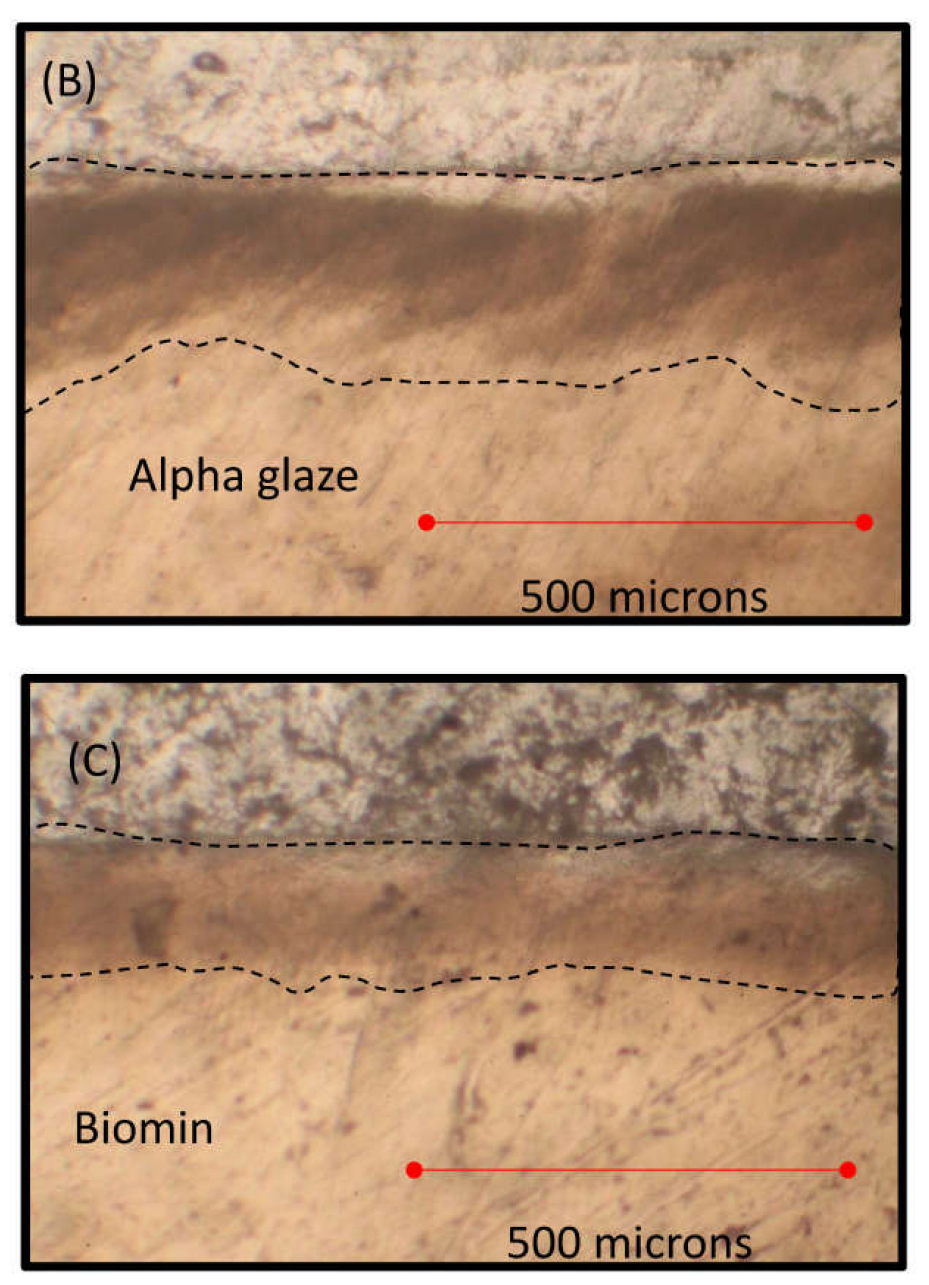
The area bound between the two dotted lines represents the subsurface enamel demineralized lesion treated by the agents employed in this study. (**A**) Lesion area formed in the control group. (**B**) Lesion area formed in the Alpha-Glaze group. (**C**) lesion area formed in the Biomin group.

**Figure 4:**
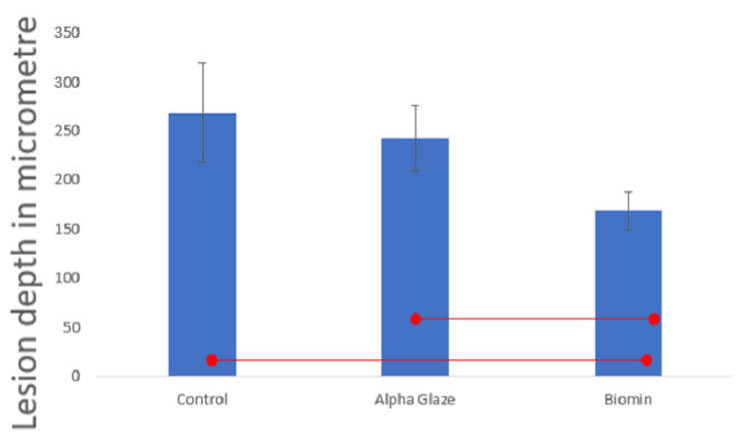
Lesion depth in microns. Significant values are connected by lines *p* < 0.05.

There was no statistically significant difference between the control and Alpha-Glaze in lesion depth. However, the FBAG showed significant less lesion depth Figure 3 and Figure 4.

## 4. Discussion

The null hypotheses adopted in the current study were partially rejected:

1. There was no negative effect on the shear bond strength of Transbond XT to tooth structure after application of the fluoride bioactive glass or Alpha-Glaze sealer onto the enamel surface when compared to Transbond XT.
2. Brushing abrasion affected the fluoride bioactive glass formed layer when compared to the Alpha-Glaze resin sealer layer.
3. The fluoride bioactive glass exerted a more protective effect on enamel when compared to the Alpha-Glaze sealer.

The fluoride bioactive glass and Alpha-Glaze sealer did not significantly affect the shear bond strength of Transbond XT to the enamel surface. However, the brushing abrasion resistance after the application of the fluoride bioactive glass was less than the Alpha-Glaze sealer, and Alpha-Glaze did not protect the enamel from demineralization compared to the fluoride bioactive glass.

In the current study, we adopted a shear bond strength test to mimic a clinical situation for the orthodontic debonding procedure [20,23]. The current results show no significant statistical difference in the shear bond strength between the three groups.

Regarding the Adhesive Remnant Index observations, 40% of the Alpha-Glaze specimens scored (3) and 6% scored (4), which may be attributed to phosphoric acid etching enamel and the concomitant penetration of resin within the enamel structure leading to the formation of resin tags within the enamel structure. During the orthodontic brackets debonding procedure, these resin tags will be detached from the enamel and may cause enamel damage of varying degrees [24,25].

On the other hand, the FBAG group showed no cohesive failure within the enamel and the Adhesive Remanent Index for this group scored (1) in 60% of its specimens. Previous studies showed that FBAG is associated with forming the interaction layer, which is mainly composed of a few micron thickness layers rich in calcium and phosphate [17,18,26,27]. This layer might have diminished the etching effect of the phosphoric acid on enamel, leading to less resin tag formation [25] and less chance of enamel damage [28].

A brushing abrasion challenge test was also conducted in the current study to evaluate the durability of the suggested orthodontic sealants tested. Since comprehensive fixed orthodontic treatment typically does not last for less than a year, a brushing cycle of 7500 was chosen in our experiment as it corresponded to one year of brushing [21]. An excellent orthodontic sealer is expected to be in place for the entire treatment with an insignificant requirement for reapplication. In the brushing abrasion challenge experiment, the Alpha-Glaze resin sealer group scored (3), which suggests that micromechanical interlocking between the Alpha-Glaze resin and enamel prevented the dislodgment of the resin particles from the enamel [29]. FBAG scored (1) showing less abrasion resistance when compared to the Alpha-Glaze [17,26,27]. However, this experiment was conducted using a stereomicroscope with ×10 magnification, which may have not detected the whole remnant of the FBAG which was previously reported to be approximately 15–70 um [17,18,26,27].

Transmitted light microscopy examination showed that the FBAG group recorded the least lesion depth when compared to the other two groups, suggesting that the FBAG formed an “interaction layer” of hydroxyapatite crystals on the enamel surface that are rich in calcium and phosphate [17,26,27]. This layer might have formed a barrier of acid-resistant crystals on top of the enamel surface; moreover, it could be suggested that parts of this calcium–phosphate-rich layer might have dissolved in the low-pH acidity of the demineralization solution, resulting in diminishing its acidity [30]. On the other hand, the Alpha-Glaze resin group did not show significant protection of the enamel subsurface, which may be attributed to possible degradation of the surrounding silane coupling agent or resin matrix and loss of filler particles [31]. Additionally, the HEMA and Bis-GMA contained in the Alpha-Glaze might have exhibited hydrolysis [29,32,33,34,35], leading to penetration of the acidic solution within the structure of the Alpha-Glaze reaching the enamel and causing its demineralization. 

**Figure 5:**
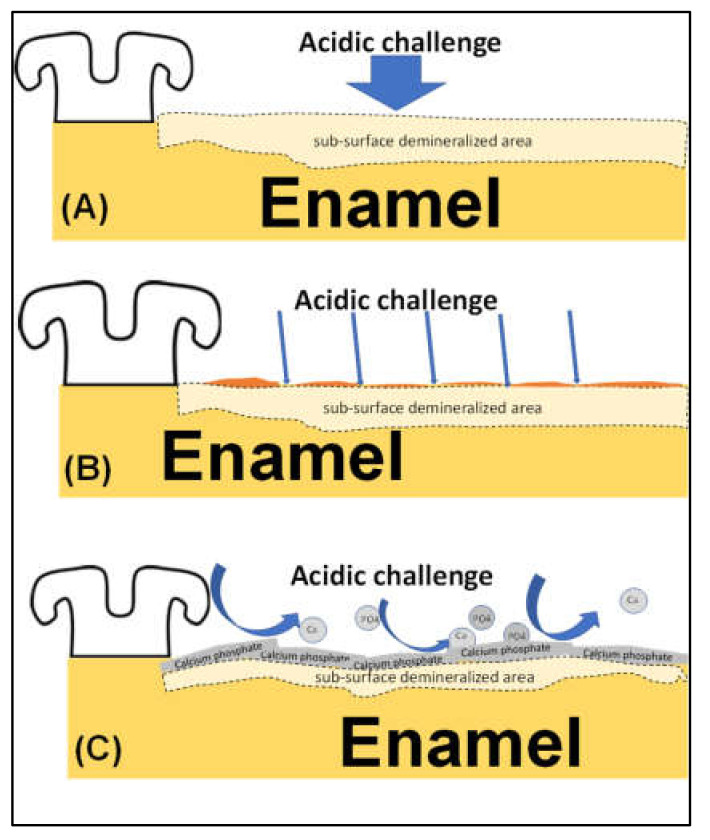
Suggested mechanism of action for the tested materials. (**A**) Control group: untreated enamel surface exposed to acidic challenge formed a sub-surface demineralized area. (**B**) Alpha-Glaze group: the enamel surface coated by Alpha-Glaze when exposed to acidic challenge showed partial dissolution that exposed the underlying enamel to demineralization forming a sub-surface demineralized area. (**C**) FBAG group: the biomin application on enamel surface formed a calcium phosphate-rich layer onto the enamel surface that resisted the demineralization and released calcium and phosphate ions when exposed to acidic challenge. Enamel was less affected by the demineralization challenge.

Our hypothesis is supported by previous research that showed no significant protective effect of Alpha-Glaze on enamel when exposed to an artificial tooth decay challenge [36]. The suggested mechanism of action for the tested materials is demonstrated in Figure 5. It is worth mentioning here that, although there was an apparent advantage in using the Alpha-Glaze seal as a sealant due to its light-curing matrix that can polymerize within few seconds of application, the light-curing matrix of this product was vulnerable to dissolution by low-pH liquid media. However, significantly better protection for the enamel was observed when using the FBAG paste, which had a retarded setting time and needed a temporary coverage by light-curing resin. Thus, it is suggested that research should be conducted to simplify the application mode of FBAG paste. The limitations of the currently presented work might include comparing two materials with different compositions and mechanisms of action as illustrated in Figure 5. Moreover, the FBAG paste relies on its bioactive action (that needs 24 h), which is enhanced by adding diluted phosphoric acid, while the Alpha-Glaze is a photo-curing resin that polymerizes within a few seconds to allow immediate protection to enamel surfaces bonded by orthodontic brackets. Future studies should be implemented to compare different bioactive materials with our suggested FBAG paste to verify the exact potency of our material compared to materials with a close similarity in their chemical composition and mechanism of action.

## 5. Conclusions

Fluoride bioactive glass (FBAG) paste can serve as an orthodontic sealer that would not affect the shear bond strength of orthodontic brackets to enamel and cause minimal enamel damage during orthodontic bracket debonding. However, its relatively long application duration poses some clinical difficulty. Alpha-Glaze resin sealer provides a mechanical barrier onto enamel surface within seconds after its polymerization, however, it did not show significant protection to enamel from a simulated acidic cariogenic challenge.

## Figures and Tables

**Table 1:** Composition of the material used in this study.

Product	Composition
BioMinF^®^, fluoride bioactive glass (FBAG) (Wako, Osaka, Japan)	(22–24 mol % Na_2_O, 28–30 mol % CaO, 4–6 mol % P_2_O_5_, 36–40 mol % SiO_2_, and 1.5–3.0 mol % CaF_2_)
2. Alpha-Glaze^®^ (resin sealer) (Dental Technologies Inc., Lincolnwood, IL, USA)	BIS-GMA 40–50%, Blend of multifunctional Methacrylates 30–40%, THF Methacrylate 20–30%, Photo initiator < 1% Stabilizer < 1%
3. Transbond XT^®^ (control) (3M Unitek, Monrovia, CA, USA)	Silane-treated quartz, (Bisgma), (2-hydroxyethyl ether), Silane-treated silica

**Table 2:** Surface preparation and Groupings.

Group	Enamel Surface Preparation	Number
FBAG	Biomin + 35% phosphoric acid + 35% Phosphoric acid + Transbond XT primer + Transbond XT adhesive	Total N = 45 Shear Bond Strength Test = 15 Brushing Abrasion Challenge Test = 15 Transmitted Light Microscopy Examination = 15
Alpha-Glaze sealer	35% Phosphoric acid + Alpha-Glaze sealer Transbond XT primer + Transbond XT adhesive	Total N = 45 Shear Bond Strength Test = 15 Brushing Abrasion Challenge Test = 15 Transmitted Light Microscopy Examination = 15
Transbond XT (Control)	35% Phosphoric acid + Transbond XT primer + Transbond XT adhesive	Total N = 45 Shear Bond Strength Test =15 Brushing Abrasion Challenge Test = 15 Transmitted Light Microscopy Examination = 15

**Table 3:** Description of the Adhesive Remnant Index (ARI) scores.

Score	Reading
0	No adhesive remanent on the tooth surface
1	If there was less than 50% adhesive remanent on the tooth surface
2	If there is more than 50% adhesive remanent on the tooth surface
3	100% of the adhesive remanent with a visible bracket impression
4	Any of the enamel chipping off could be detected

**Table 4:** The distribution of means and standard deviations of shear bond strength (SBS) in MPa, with results of post hoc test.

Material	Shear Bond Strength	
FBAG group	(28.1 ± 5.5 Mpa)	
Alpha-Glaze group	(32.5 ± 7.4 Mpa)	
Transbond XT (Control)	(30.7 ± 6.5 Mpa)	
Post Hoc Comparisons		
		p_tukey_
Alpha-Glaze	FBAG	0.264
Alpha-Glaze	TransBond XT	0.774
FBAG	TransBond XT	0.632

**Table 5:** ARI score distribution after shear bond strength.

Score	FBAG	Alpha-Glaze	Transbond XT (Control)
Score 0 No adhesive left on the tooth	6 40%	3 20%	5 33.30%
Score 1 Less than 50% adhesive left on the tooth	9 60%	5 33.3%	5 33.3%
Score 2 More than 50% adhesive left on the tooth			4 26.6%
Score 3 100% of the adhesive left on the tooth		6 40%	
Score 4 If any enamel chipping off could be detected		1 6.6%	1 6.6%

**Table 6:** ARI score distribution after brushing abrasion challenge test.

Score	FBAG	Alpha-Glaze
Score 0 No material left on the tooth		
Score 1 Less than 50% of the material left on the tooth	100%	
Score 2 More than 50% of the material left on the tooth		
Score 3 All of the material left on the tooth		100%

## Data Availability

Available upon request from the corresponding author.
